# A Gene Expression Signature for RSV: Clinical Implications and Limitations

**DOI:** 10.1371/journal.pmed.1001550

**Published:** 2013-11-12

**Authors:** Peter J. M. Openshaw

**Affiliations:** National Heart and Lung Institute, Imperial College, London, United Kingdom

## Abstract

Peter Openshaw discusses the challenges in advancing respiratory syncytial virus (RSV) treatments and the implications of a study by Mejias and colleagues using a newly identified gene signature for diagnosis and prediction of RSV severity.

*Please see later in the article for the Editors' Summary*

**Linked Research Article**

This Perspective discusses the following new study published in *PLOS Medicine*:

Mejias A, Dimo B, Suarez NM, Garcia C, Suarez-Arrabal MC, et al. (2013) Whole Blood Gene Expression Profiles to Assess Pathogenesis and Disease Severity in Infants with Respiratory Syncytial Virus Infection. PLoS Med 10(11): e1001549. doi:10.1371/journal.pmed.1001549.

In this study, Mejias and colleagues found that specific blood RNA profiles of infants with RSV LRTI allowed for specific diagnosis, better understanding of disease pathogenesis, and better assessment of disease severity.

Especially during the first year of life, children are plagued by pathogens jockeying for position at the mucosal surfaces [1]. Respiratory viruses have diverse pathways to success: influenza is highly infectious and undergoes rapid evolution to escape protective immunity, while rhinoviruses succeed by being so diverse that we are unlikely to experience all of them in a lifetime. With both these viruses, reinfection with the exact same strain is exceptional.

## Immune Responses to RSV Infection

With respiratory syncytial virus (RSV), however, the situation is curiously different. RSV shows antigenic variation, but the weak protective immune response induced by natural infection wanes rapidly, especially in the upper respiratory tract. Even antigenically similar or identical viruses are able to reinfect with relative ease. How RSV gets around our immune defences continues to puzzle immunologists [2].

Although RSV usually causes relatively trivial common cold symptoms, it can also have serious and devastating effects in vulnerable populations. Worldwide, it is a major source of disease and death in children under 5 years of age [3] and is increasingly appreciated as a cause of significant winter morbidity and mortality amongst elderly [4],[5] and immunocompromised people. In addition, delaying RSV disease by passive antibody therapy with antiviral monoclonal antibodies palivizumab not only prevents bronchiolitis, but also causes a remarkable reduction in the frequency of recurrent wheeze in later childhood [6].

While many infectious diseases can be prevented by vaccination, over 40 years of work has failed to produce a vaccine for RSV disease. The field is now highly active again, with many vaccine producers seeing RSV as one of the major large unmet needs. While on-going investments in vaccine research raise hope that a vaccine for RSV might become available in the next 5 years, it remains unclear what type of immune response is most protective against infection, or how to be sure that a vaccine will not enhance disease.

## The Uses of Whole Blood RNA Analysis

The technique of whole blood RNA profiling is now well established as a research tool. The RNA obtained is predominantly cellular, although serum does contain small amounts of intact mRNA and micro (mi)RNA bound to protein, HDL, or in apoptotic debris [7]. Whole blood RNA profiling is certainly more than just an expensive blood count: results depend not only on the number and type of cells in the blood sample, but also on the activation state and the exposure of the cells to stimulatory or inhibitory factors in the blood or in the tissues through which the cells have passed. The circulating cells may represent cells in transit from one site to another, overspill from tissue inflammation, or may be cells that have been rejected by the tissue as irrelevant.

In this context, the recent study by Asuncion Mejias and colleagues in *PLOS Medicine* represents a significant advance in our understanding of RSV's special relationship with the immune system. By analysing the transcriptional profile from the blood of children infected with RSV, influenza, or rhinovirus, the authors aim to identify biomarkers that both aid in diagnosis and predict severity. Immunologists will be especially excited to see gene clusters changing in ways that correlate with the nature of the infecting pathogen, giving clues about pathogenesis and the protective mechanisms that operate differently in the response to diverse infections.

## What Does It Tell Us about RSV Disease?

RSV disease can be regarded as a dysregulated immune response to viral infection [2]. Interestingly, Mejias and colleagues found that RSV infection was associated with neutrophil activation (a cell type known to contribute to RSV disease [8]), increased markers of inflammation, and upregulation of interferon-related genes, but with suppression of T and B cell responses. This pattern was especially marked in infants aged less than 6 months. Although the transcriptomic profile was assessed relatively late in disease (typically 5–6 days after symptom onset), it still helped anticipate subsequent outcome.

This study moves the field several steps towards the clinical use of transcriptomic profiling in the diagnosis and prognostication of children with acute respiratory infections. The finding that innate inflammatory genes are upregulated during RSV infection emphasises the importance of mucosal inflammation, and fits well with previous findings from genetic studies that highlight innate immunity in RSV disease [9].

The reduction in genes normally associated with T and B cells has alternative interpretations. These cells might be depleted from peripheral blood because they are accumulating at the site of disease; in this case, their absence from blood demonstrates their importance in the pathogenesis. Alternatively, it could be that part of RSV's evasion strategy is to inhibit these responses, destroying the cells that are vital for protection against reinfection. This second possibility is supported by studies showing a lack of T cells in the lungs of children with bronchiolitis [10], but this remains controversial.

Another response that is conspicuously absent is the T-helper 2 pattern (IL-4, IL-5, IL-13) often associated with asthmatic or atopic responses. Some studies of the pulmonary response to RSV indicate an over expression of such genes and an increase in Th2 cytokines [11], but no such signature appears in the studies of Mejias and colleagues. Here again, the peripheral blood may not be telling the whole story and needs to be complemented by detailed studies of the response in the respiratory tract.

The findings with respect to the activation of type 1 interferon genes are intriguing. It has long been known that the non-structural proteins of RSV, expressed in great abundance in the earliest phase of infection, are capable of inhibiting host interferon responses [12]. However, the present study shows a significant over-expression of interferon-related genes, especially in children with severe illness. It is important to acknowledge that the children were only enrolled into the study relatively late in disease; it will be interesting in future studies to see which patterns of gene expression are present earlier in disease, at a time when viral load may be greatest and the potential for viral inhibition of interferon is greater. However, Mejias and colleagues found that the interferon activation signal increases still further during convalescence, raising the possibility that active virus replication does indeed partially inhibit interferon responses and that this inhibition lifts during viral clearance.

## What Does the Future Hold?

Although expression profiling of cells in the peripheral blood has limitations in terms of monitoring the responses in the affected tissue, the technique clearly has great promise for discovering combinations of gene expression that are indicative of both specific pathogens and also the future course of disease.

With multiplex testing for combinations of disease markers that indicate disease subtypes (or “endophenotypes”) becoming ever more feasible as a near-patient diagnostic aid, studies such as that by Mejias and colleagues may give rise to new and informative tests that help to define the pathogenesis of disease on an individual basis and thus dictate clinical management. This is especially important in an era in which many new drugs are biological agents, targeting specific disease pathways that may be important in some patients but not others.

Clinicians will hope that improved diagnostic accuracy from multiplex tests arising from broad gene expression screens will be accompanied by the introduction of such new treatments. The golden combination of rational testing linked to specific treatment should allow avoidance of expensive or harmful interventions, streamline the care of vulnerable patients, and at last provide new ways to treat RSV disease that go beyond the simply supportive. The study from Mejias and colleagues is a significant step towards that long-awaited goal.

## Abbreviations

RSV: respiratory syncytial virus
